# Estimating HIV incidence in the Akwa Ibom AIDS indicator survey (AKAIS), Nigeria using the limiting antigen avidity recency assay

**DOI:** 10.1002/jia2.25669

**Published:** 2021-02-22

**Authors:** Olubunmi R Negedu‐Momoh, Oluseyi Balogun, Ibrahim Dafa, Akan Etuk, Edward Adekola Oladele, Oluwasanmi Adedokun, Ezekiel James, Satish R Pandey, Hadiza Khamofu, Titi Badru, Janet Robinson, Timothy D Mastro, Kwasi Torpey

**Affiliations:** ^1^ Laboratory services and HSS Department FHI 360 Abuja Nigeria; ^2^ Global Public Health Department IHR Strengthening Programme Public Health England Abuja Nigeria; ^3^ Laboratory Services Department University of Uyo Teaching Hospital Uyo Nigeria; ^4^ Prevention, Care and Treatment Department FHI 360 Abuja Nigeria; ^5^ Monitoring and Evaluation Department FHI 360 Abuja Nigeria; ^6^ Office of the HIV/AIDS and TB United States Agency for International Development (USAID) Abuja Nigeria; ^7^ Program Management Department FHI 360 Abuja Nigeria; ^8^ Infectious Diseases and Health Systems FHI 360 Durham NC USA; ^9^ FHI 360 Durham NC USA; ^10^ College of Health Sciences University of Ghana Accra Ghana

**Keywords:** HIV‐1, incidence, recent infection, limiting antigen avidity, viral load, Nigeria

## Abstract

**Introduction:**

HIV incidence estimates are important to characterize the status of an epidemic, identify locations and populations at high risk and to guide and evaluate HIV prevention interventions. We used the limiting antigen avidity assay (LAg) as part of a recent infection testing algorithm to estimate HIV incidence in the Akwa Ibom AIDS Indicator Survey (AKAIS), Nigeria.

**Methods:**

In 2017, AKAIS, a cross‐sectional population‐based study was conducted at the household (HH) level in 31 local government areas (LGAs) of Akwa Ibom state. Of the 8963 participants aged ≥15 years who were administered questionnaires for demographic and behavioural data, 8306 consented to HIV rapid testing. Whole‐blood specimens were collected from 394 preliminary HIV‐seropositive individuals for CD4+ cell count determination and plasma storage. Samples were shipped to a central quality laboratory for HIV confirmatory testing and viral load determination. A total of 370 HIV‐positive specimens were tested for the recent HIV infection using the LAg assay.

**Results:**

Of the 8306 consenting adults, the HIV prevalence was 4.8%. Of the 370 HIV‐positive samples tested for HIV recency, the median age was 35 years, 48.8% had CD4+ cell count >500/mm^3^ and 81.3% was not virally suppressed. Viral suppression was greater among females (21%) than for males (13%). A total of 11 specimens were classified as recent based on the LAg assay and HIV viral load ≥1000 copies/mL. The weighted, adjusted HIV‐1 incidence was 0.41/100 person‐years (95% CI 0.16 to 0.66); translating to 13,000 new cases of HIV infections annually in Akwa Ibom, a state with a population of 5.5 million. The HIV incidence rate was similar in females and males (0.41% and 0.42% respectively). The incidence rate was the highest among participants aged 15 to 49 years (0.44%, 95% CI 0.15 to 0.74) translating to 11,000 new infections annually, about 85% of all new infections in the state.

**Conclusions:**

The finding of the high HIV incidence among the 15 to 49‐year age group calls for renewed and innovative efforts to prevent HIV infection among young adults in Akwa Ibom state.

## 1 INTRODUCTION

HIV incidence reflects the rate of new infections over time in a population [1]. The measurement of HIV incidence can help assess the status of an epidemic, identify populations subgroups and geographical settings at increased risk to target programmes and measure the effectiveness of prevention interventions. For more than 20 years, the use of a serological assay that can distinguish recent from long‐standing HIV infection has been used to estimate HIV incidence from cross‐sectional surveys [2, 3, 4]. Several assays that classify HIV‐reactive specimens have been developed or adapted to distinguish recent or long‐term infected persons based on the maturation of the immune response. However, the development of HIV incidence assays has been challenging due to factors such as variability in immune responses at an individual and population level, variability by HIV‐1 subtype, access to antiretroviral therapy, decreases in the genetic diversity of the HIV in the era of antiretroviral therapy, advanced HIV disease and other factors that are not well understood that can lead to misclassification of individuals [5, 6].

Akwa Ibom State of Nigeria, with a population of 5.5 million, was selected by the United States President’s Emergency Plan for AIDS Relief (PEPFAR) for an HIV population‐based survey called Akwa Ibom AIDS Indicator Survey (AKAIS), with support from United States Agency for International Development (USAID). The study was designed to fill some gaps in HIV data, including HIV incidence, as well as to provide evidence‐based guidance to the design of future HIV control activities in the State and Nigeria. AKAIS was adapted from the CDC’s HIV Impact Assessments (HIAs) and serves as a model for other Nigerian states HIV‐focused population‐based studies with comprehensive biological testing. AKAIS is the first HIV incidence laboratory measurement study conducted in Nigeria.

In this study, we describe the detection of recent HIV‐1 infection using the conventional limiting‐antigen avidity enzyme immunoassay (LAg‐Avidity EIA) in the Akwa Ibom state cross‐sectional population‐based survey (AKAIS). The Mahiane synthetic cohort approach for estimating age‐specific incidence was applied for comparison.

## 2 METHODS

### 2.1 Ethical approval

Ethical approval for the study protocol was received from the FHI 360 Protection of Human Subjects Committee (institutional review board), North Carolina, U. S., the Akwa Ibom State Ministry of Health Ethics Committee, the University of Uyo Teaching Hospital Review Committee and the University of Nigeria Nsukka Teaching Hospital Health Research Ethics Review Committee. Written informed consent was obtained from the study participants before enrolment.

### 2.2 Study setting

AKAIS population‐based cross‐sectional household (HH) study was conducted in all 31 Local Government Areas of Akwa Ibom state from 4313 HH from April to June 2017. Of the 16,994 people who were listed for the survey, 16,288 completed the interviews including the informed and written consent (8963 adults aged 15 years and older). Of the 8963 individuals, specimen collection, and screening for HIV were completed for 8306 persons [7]. Participants aged 15 to 17 years who were considered mature minors were allowed to consent for themselves, whereas parental or guardian consent and minor assent were required for participation in the interview and blood draw for other minors of the same age group. Electronic data capture into an android‐based portable tablet was employed using Census and Survey Processing System (CSPro) software. Barcodes with unique identification numbers were generated for the consenting participants. Barcode was scanned using a barcode reader into the tablet and questionnaires were administered to participants to obtain demographic and behavioural data such as reproductive history, marital status, sexual activity, fertility, family planning and other health issues, in settings which afforded privacy. Barcoded stickers were placed on the consent forms, questionnaires, sample bottles, sample tracking forms.

Laboratory mapping was done to assign satellite laboratories within the local government areas (LGAs) that linked to the central quality control laboratory of the University of Uyo Teaching Hospital. Eleven satellite laboratories were selected based on the availability of the BD FACSCount flow cytometer, human resource, power supply, storage facility and proximity to the enumeration area sample of clusters for the household.

### 2.3 Field data management

Data generated using CSPro software were transferred from survey team members to the central server. The CSPro software template has all questionnaire modules in the tablet as applicable from the field to the laboratory entry. Barcodes with Unique Specimen Identification numbers were printed in real‐time within the households by the survey team and placed on laboratory specimens. The barcode information was also placed on the individual consent forms and entered into the participants’ data to enable accurate merging of laboratory (biological) data and individual questionnaire (behavioural) data.

### 2.4 HIV testing and laboratory methods

Rapid HIV‐1/2 testing using finger‐prick blood was performed in the household on 8306 consenting adults by following the national serial guidelines for the HIV testing algorithm (Determine – Unigold – Stat‐Pak) [8, 9] (see additional file S1). Whole‐blood specimens were collected from 394 HIV‐positive individuals into 10 mL ethylenediaminetetraacetic acid (EDTA) vacutainer tubes at the household and sent to satellite laboratories. CD4 cell count testing was carried out on the HIV‐positive specimens using the BD FACSCount flow cytometer within six hours of sample collection according to the standard operating procedure [10].

### 2.5 Sample processing at the satellite laboratory

Following the CD4 testing, whole‐blood samples were processed into plasma by centrifuging at 1600 *g* for 20 minutes at room temperature. 1.2 mL of plasma each was then transferred into two pre‐labelled sterile 2.0 mL polypropylene screw cap tubes (cryovials), whereas 0.5 mL plasma each was transferred into two other pre‐labelled cryovials for HIV Incidence test and 100µl plasma to other pre‐labelled cryovials for a confirmatory test. Pre‐printed barcode numbers (unique identifiers) were placed on the pre‐labelled cryovials also with the sample transfer form. Aliquoted plasma was then stored at 2°C to 8°C within two days or frozen at −20°C until transported in appropriate condition to UUTH central quality laboratory.

### 2.6 Sample processing at the central quality laboratory

The samples were transported to the Central Quality Laboratory of the University of Uyo Teaching Hospital, Uyo for HIV confirmatory test using Biorad Geenius HIV 1/2 kit (Biorad, France) [11]. The flow is detailed in additional file S1. The confirmed HIV‐seropositive samples were further tested for HIV‐1 RNA viral load using Cobas Roche Ampliprep/Taqman analyser version 2.0 on the 96 CAP‐ CTM analyser (COBAS AmpliPrep/COBAS Taqman HIV‐1 Quantitative Test, v2.0, Roche Diagnostic GmbH, Germany) in line with manufacturer instructions using plasma aliquot of 1.0 mL [12]. Viral suppression was taken as ≤1000 copies per mL based on WHO guidelines [13].

### 2.7 HIV incidence estimates using a recent infection testing algorithm

**Figure 1:**
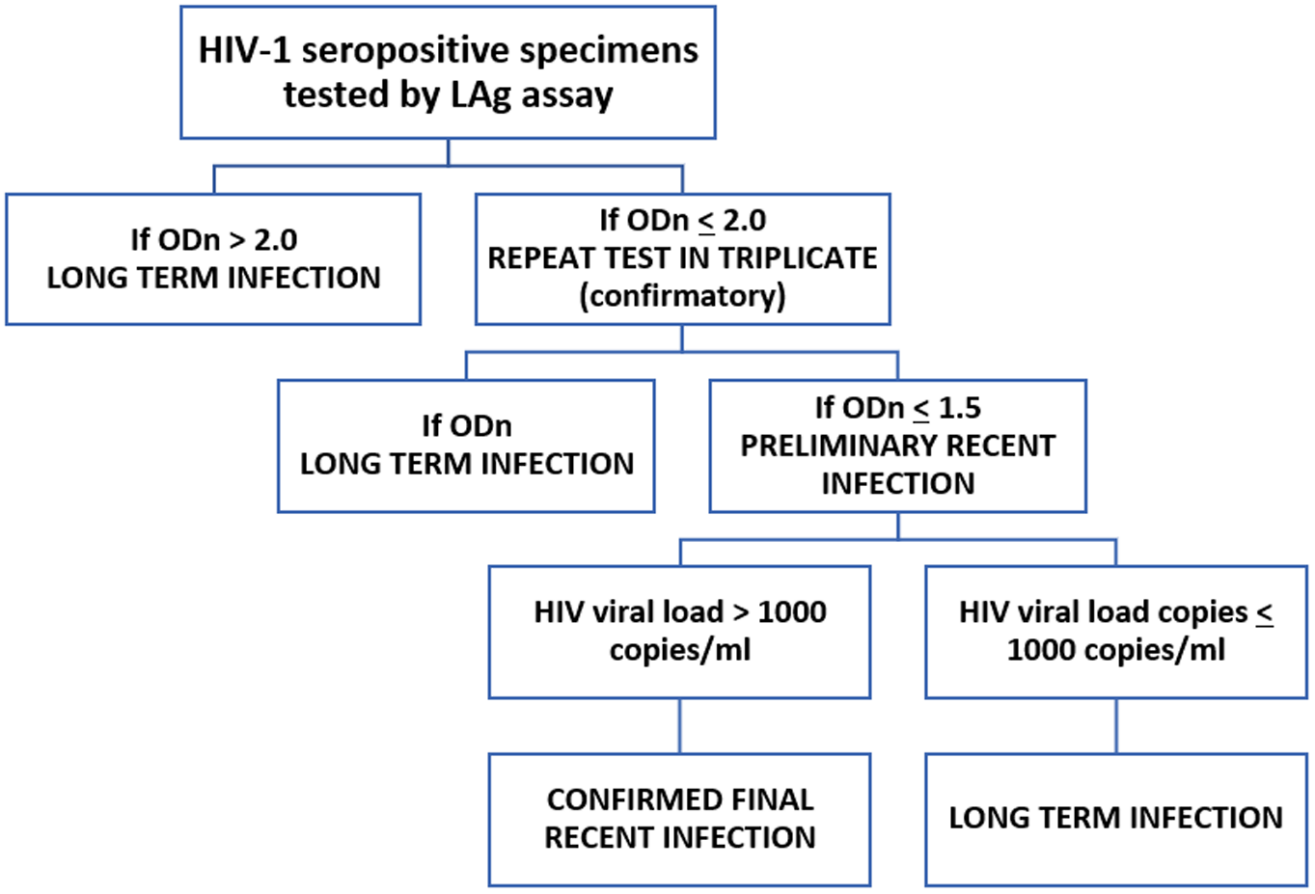
Recent infection testing algorithm (RITA). (The RITA involves the use of new Limiting avidity (LAg) incidence assay combined with additional laboratory (e.g. viral load data that can be used to classify HIV infections as recent or long‐term HIV infection).

A total of 370 eligible plasma samples were tested for recent HIV infection using Sedia Limiting Antigen Avidity Assay kit (LAg) (Sedia Bioscience Inc. Portland Oregon, USA) with a cutoff normalized optical density (ODn) of 1.5 [2, 14]. HIV incidence was estimated using a recent infection testing algorithm (RITA), the Sedia LAg data management sheet (see additional file S2) and HIV incidence calculator developed by CDC and recommended by the WHO Incidence Working Group and the Consortium for Evaluation and Performance of Incidence Assays on Global HIV/AIDS and STI Surveillance [15, 16, 17]. The conventional biomarker for a recent infection testing algorithm uses the LAg‐Avidity assay combined with an HIV viral load assay (Figure 1). The viral load cut off of >1000 copies/mL was applied to minimize potential misclassification of the LAg assay thereby excluding “false recent” results [2, 14]. A false‐recent rate (FRR) of 0.0% was applied as there was no baseline FRR available for the country at the time of study. HIV‐incidence calculation took into account the complex sampling design and weighted numbers in the incidence formula. The mean duration of recent infection of 130 days was applied in the incidence calculation [15, 18]. The MDRI and FRR used for the AKAIS study were decided by an interagency process among CDC, USAID and FMOH following the UNAID/WHO 2015 to assure comparable results as MDRI were not available for the circulating subtype in Nigeria [19].

### 2.8 HIV Incidence estimates using age‐specific incidence of Mahiane *et al*. “synthetic cohort” method

Age‐specific incidence was estimated from the age structure of prevalence using the Mahiane synthetic cohort estimator [20] The study assumes a stable epidemic where the age structure of prevalence is not changing at a significant rate in secular time to allow for using a single cross‐sectional prevalence survey to estimate age‐specific incidence as suggested by Grebe *et al* [21].

The age‐specific incidence is given by.

$$\lambda \left(a\right)=\left(\frac{1}{1‐p\left(a\right)}\right)\frac{d}{\mathrm{dp}}p\left(a\right)+\delta \left(a\right)p\left(a\right).$$

where $p\left(a\right)=\frac{1}{1+\exp\left(‐\beta_{0}‐\beta_{1}a‐\beta_{2}a^{2}‐\beta_{3}a^{3}\right)}$ is the age‐specific prevalence, $\delta \left(a\right)$ is the age‐specific excess mortality and the derivative of HIV prevalence with respect to age is given by

$$\frac{d}{\mathrm{da}}p\left(a\right)=\frac{\exp\left(\beta_{0}+\beta_{1}a+\beta_{2}a^{2}+\beta_{3}a^{3}\right)\times \left(\beta_{1}+2\beta_{2}a+3\beta_{3}a^{2}\right)}{{\left(1+\exp\left(\beta_{0}+\beta_{1}a+\beta_{2}a^{2}+\beta_{3}a^{3}\right)\right)}^{2}}$$

The standard error and the 95% confidence interval of all incidence estimates were estimated using bootstrapping with 1000 replication. Data management and analysis was conducted using Stata 16 (StataCorp, College Station, TX, USA).

## 3 RESULTS

**Figure 2:**
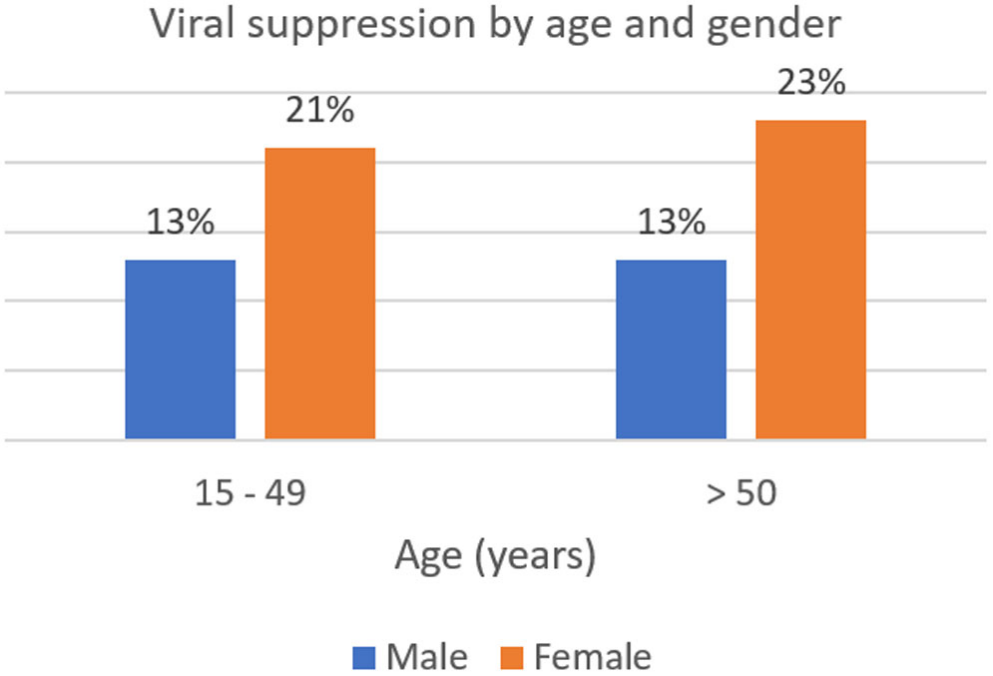
Viral suppression by gender among the age groups.

**Figure 3:**
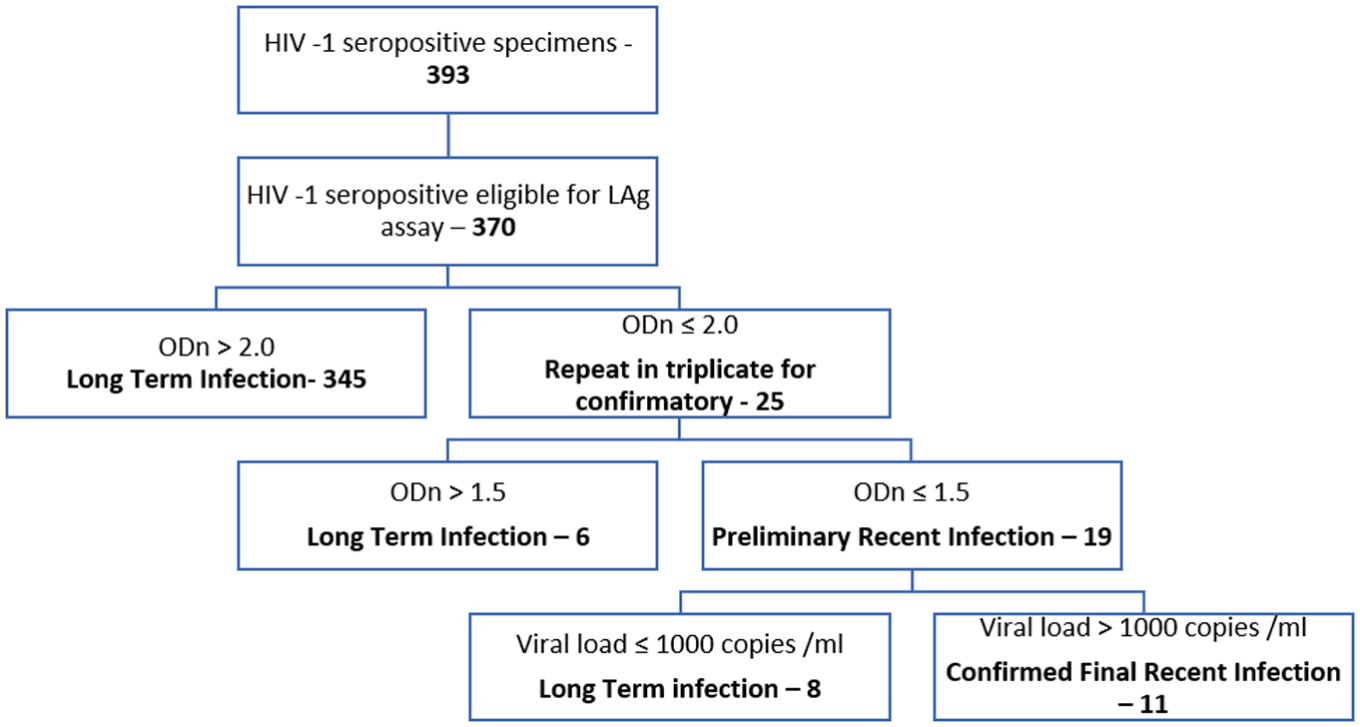
Flow chart of samples categorized according to the Recent Infection Testing Algorithm.

Of the 8306 consenting adults aged 15 years and older tested, 394 tested positive (393 HIV‐1: 1 HIV‐2) with a point prevalence of HIV at 4.8% (females: 5.6%; 95% CI 4.9 to 6.4 and males: 3.7%; 95% CI 3.0 to 4.4). Of the 370 HIV‐1‐seropositive samples tested for HIV recency, the median age was 35 years, 48.8% had CD4+ cell count >500/mm^3^ and 81.3% were not virally suppressed (Table 1); viral suppression was relatively better among female than male within the age 15 to 49 years (females 21%, males 13%) and >50 years (females 23%, males 13%) (Figure 2). A total of 19 samples were identified as LAg EIA preliminary recent, 351 as long‐term infection. Applying the conventional biomarker for recent infection, eleven of the LAg EIA recent specimen with viral load >1000 copies/mL were classified as recent, whereas eight (8) with viral load copies <1000 copies/mL were long‐term infections that are found in the drug‐experienced patient with low viral load infection and the elite suppressors (Figure 3) (see additional file S2). The weighted unadjusted HIV‐1 incidence estimate was 0.72/100 PY, whereas the adjusted HIV‐1 incidence after viral load exclusion (<1000 copies/mL) was 0.41/100 PY.

**Table 1:** Patients characteristics for HIV Incidence testing

Baseline characteristics	n (%)
Gender	
Female	243 (65.7)
Male	127 (34.3)
Age, median (IQR)	35 (28 to 45)
CD4 (cells/µL)^a^	
≤500	184 (51.2)
>500	175 (48.8)
Viral Load (copies/mL)	
Suppressed <1000	69 (18.7)
Unsuppressed ≥1000	301 (81.3)

IQR, Interquartile range.

^a^ Data available for 359 samples.

**Figure 4:**
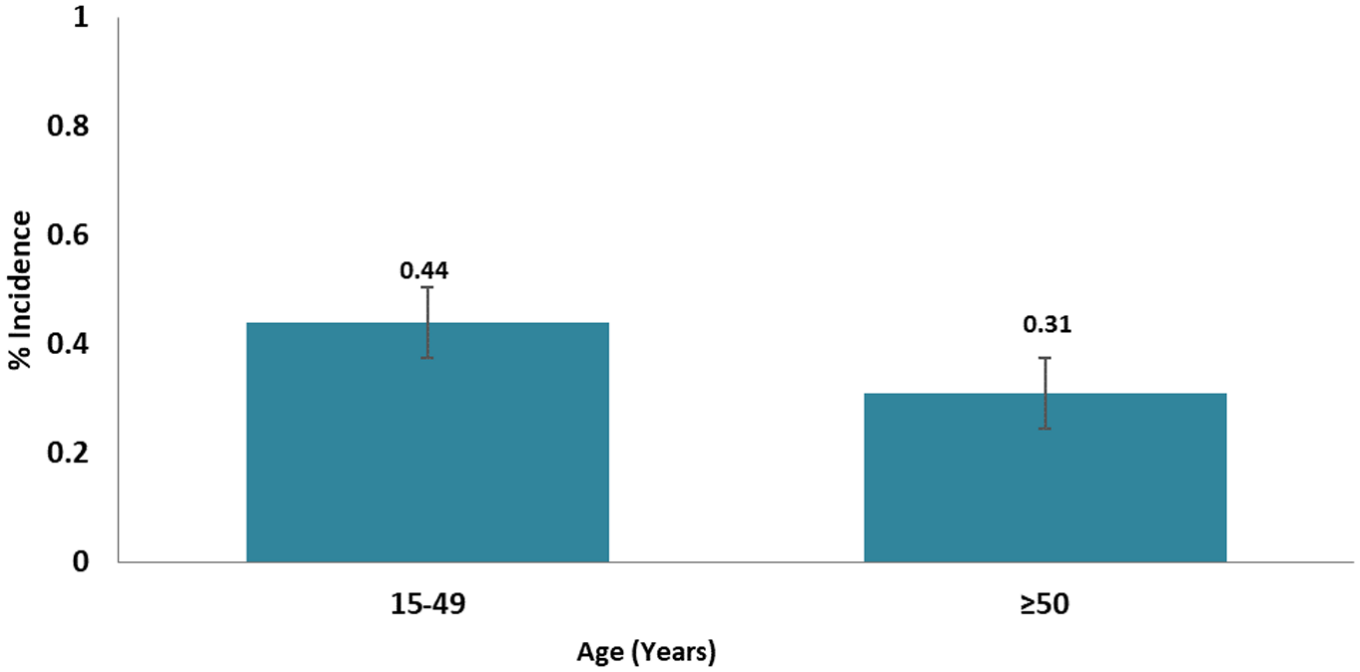
HIV incidence rate by age group using conventional biomarker of recent infection, AKAIS study.

The adjusted HIV‐1 incidence translates to 13,000 new cases of HIV infections annually in persons 15 years and older. The HIV incidence rate was similar in females and in males (females: 0.41%; 95% CI 0.08 to 0.74 and males: 0.42%; 95% CI 0.05 to 0.79). The annual incidence rate among adults ages 15 to 49 years was higher (0.44%; 95% CI 0.15 to 0.74) than age ≥50 (0.31%, 95% CI 0.00 to 0.75) (Figure 4), (Table 2), translating to 11,000 new infections in this age group and about 85% of new infections in the state population.

**Table 2:** HIV incidence (percentage) and number of new infections by age and sex among participants ages 15 years and older

	HIV incidence % (95% CI)	Estimated no. of new infections^a^
Overall	0.41 (0.16 to 0.66)	13000 (5000 to 21000)
Sex		
Male	0.42 (0.05 to 0.79)	7000 (800 to 13000)
Female	0.41 (0.08 to 0.74)	6000 (1200 to 12000)
Age group (years)		
15 to 49	0.44 (0.15 to 0.74)	11000 (3900 to 19000)
≥50	0.31 (0.00 to 0.75)	2000 (0 to 4000)

^a^ Numbers rounded off to the nearest thousand.

HIV incidence by selected behavioural and sociodemographic characteristics showed that HIV incidence among the secondary school level of education is twice compared to primary school education (0.61% vs. 0.38%). No new infections were found among those with tertiary education and without education.

HIV incidence by marital status revealed that those cohabitating with a sexual partner reported a higher incidence of 1.60% compared to married, never married and previously married people, whose rates were 0.42%, 0.42% and 0.22% incidence respectively. Respondents who reported ever having sex had an HIV incidence of 0.46%, whereas no new infections were found among those who had never had sex. Analysis of HIV incidence by place of residence revealed a slightly higher incidence rate in rural than urban areas (0.43%; 95% CI 0.13 to 0.73 vs. 0.37%; 95% CI 0.00 to 0.80). (Table 3).

**Table 3:** HIV incidence (percentage) by behavioural and sociodemographic factors among participants age 15 years and older

Variable	n (number tested by recent infection assay)	HIV incidence % (95% CI)
Level of education		
None	27	1.0 N.D
Primary	146	0.38 (0.00 to 0.82)
Secondary	159	0.61 (0.18 to 1.04)
Tertiary	22	0.00 (N.D.)
Marital status		
Never married	85	0.42 (0.00‐ 0.83)
Married	168	0.42 (0.05 to 0.80)
Previously married	95	0.22 (0.00 to 0.65)
Cohabiting	6	1.60 (0.00 to 4.68)
Sexual		
Ever had sex	351	0.46 (0.18 to 0.73)
Never had sex	3	0.0 (N. D.)
Location		
Urban	94	0.37 (0.00 to 0.80)
Rural	276	0.43 (0.13 to 0.73)

Respondent with missing values were excluded from the analysis. N. D., not determined.

**Figure 5:**
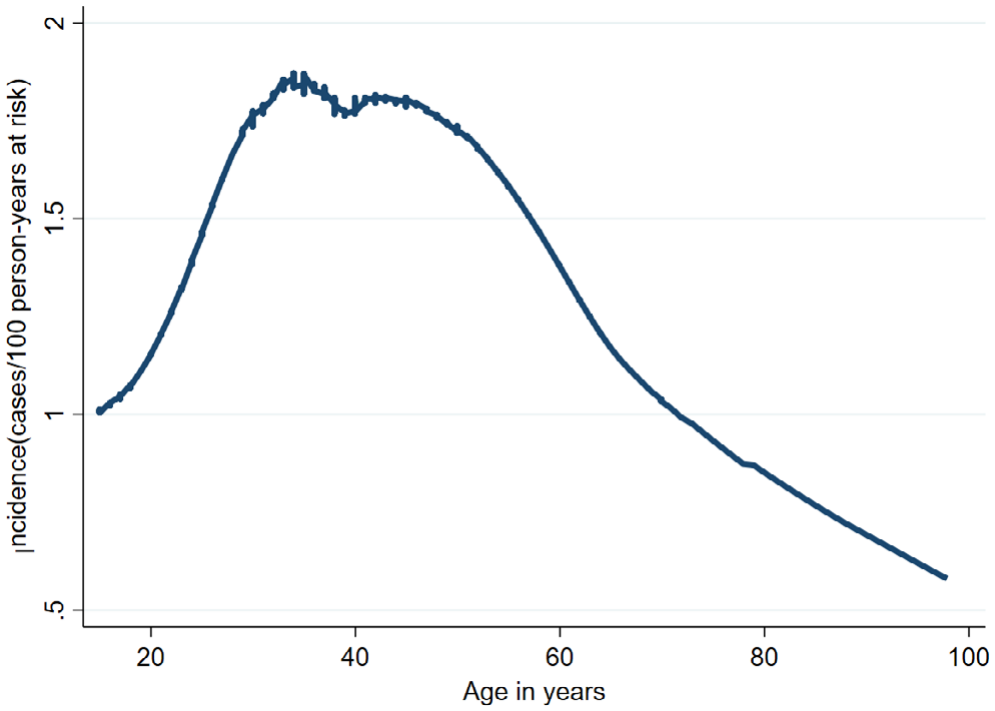
HIV incidence by age group using the Mahiane’s synthetic cohort method.

By way of comparing the Mahiane *et al*. synthetic cohort method, the overall age‐specific HIV incidence estimate for individuals aged 15 to 98 years was 1.58 cases/100 person‐years (95% CI: 1.57 to 1.59; Table 4). This incidence rate translates to 50,000 new cases of HIV infections annually in persons ≥15 years. The annual incidence rate was high in the age 15 to 49 years (1.69%; 95% CI 1.67 to 1.70) and decline after age 50 years (1.20%, 95% CI 1.19 to 1.20) (Table 4, Figure 5).

**Table 4:** Average incidence estimates by age group using Mahiane’s synthetic cohort method

Sociodemographic factors	Mahiane synthetic cohort method HIV incidence % (95% CI)	Estimated no. of new infections^a^
Overall (15 to 98)	1.58 (1.57 to 1.59)	50,000 (49400‐ 50000)
Sex		
Male	1.59 (1.58 to 1.61)	25000 (25100 to 26000)
Female	1.57 (1.55 to 1.58)]	24000 (24200 to 25000)
Age group (years)		
15 to 49	1.69 [1.67 to 1.70]	44000 (43300 to 44000)
≥50	1.20 [1.19 to 1.20]	7000 (6500 to 7000)
Location		
Rural	1.56 (1.55 to 1.58)	
Urban	1.62 (1.60 to 1.64)	

^a^ Numbers rounded off to the nearest thousand.

## 4 DISCUSSION

The difference in Akwa Ibom HIV prevalence estimated from the population‐based National AIDS and Reproductive Health (NARHS plus) survey of 2012 (6.5%) and the 2014 Antenatal Care (ANC) survey (10.8%) led to the design of the more robust AKAIS survey to provide more accurate estimates of HIV prevalence and incidence. The 2017 AKAIS study yielded an HIV prevalence of 4.8%, lower than the previous estimates and more in line with data from a national survey conducted in 2018 that found lower prevalence overall nationwide. The 2018 national survey estimated HIV prevalence in Nigeria to be 1.4% among adults aged 15 to 49 years [22, 23]. Akwa Ibom was confirmed as a relatively high HIV burden state; moreover, the incidence rate of 0.41% annually is consistent with the prevalence of 4.8% observed [7]. Achieving viral load suppression is a key to the prevention of HIV transmission. HIV viral suppression observed in this study was very low (19%); this may be due to limited knowledge of HIV status and access to treatment, despite ART scale‐up. The Joint United Nations Programme on HIV AIDS (UNAIDS) declared the 90‐90‐90 strategy expecting that 90% of those infected would know their status, of whom 90% of the positives would be on treatment and 90% of those on treatment would achieve viral suppression by the year 2020 [24]. The estimated percentage of people living with HIV in West and Central Africa who achieved viral suppression stood at 29% in 2017 and the HIV response in this region is said to lag behind the rest of sub‐Saharan Africa [25].

Earlier mathematical models of HIV incidence estimate in Akwa Ibom state using SPECTRUM software yielded annual incidence rates of 0.8% to 1.4% between 1995 and 2013 [26] Our result revealed an HIV incidence rate of 0.41% using conventional biomarkers for recent infection testing algorithm (RITA), less than one‐third of the estimate from the 2013 mathematical model of 1.4%. Our study estimated there were 13,000 new infections annually in the state.

We applied the Mahiane *et al*. synthetic cohort method to compare with our AKAIS study estimate. The incidence rate using Mahiane *et al*. method was nearly four times higher (1.58 vs. 0.41) compared to the recent infection testing algorithm (RITA) [21]. The incidence of HIV in the population was the highest in the 15 to 49 years to 1.69/100 PY. We consider the RITA‐based estimate to be more accurate considering the prevalence in the state. The high Mahiane *et al*. synthetic cohort estimate used assumptions for spectrum that could be an overestimate, probably resulting from inadequate data available for the method.

These findings highlight the importance of monitoring the burden of HIV in the wake of the call for zero new infections and the need to evaluate the current status of HIV burden in the state towards realizing the UNAIDS 90‐90‐90 targets. New HIV infections across sub‐Saharan Africa countries have declined from an estimated 2.2 million in 2005 to 0.97 million in 2019, but still accounted for 57% of the global burden [27]. In 2016, UNAID reported Nigeria alone accounted for more than half of new HIV infections in Western and Central Africa, due the Nigeria’s large population [28]. Slow progress is being made in this region in reducing new infections, especially among young people.

In Akwa Ibom, new infection in the age group 15 to 49 years was more than five times greater than age ≥50 years; this age group includes adolescents and young adults of reproductive age. This is an issue of concern with the growing population of adolescent and youth getting infected. There is a need for revised HIV programming to be more focused on this age group targeting behavioural and biomedical prevention approaches. The data also substantiate the claim that HIV is an epidemic primarily of young people and the need to reach this new generation of young vulnerable people with HIV prevention and treatment services [29, 30, 31, 32]. More research is needed to better understand the factors that are driving HIV transmission among young adults in Akwa Ibom State. Our result is consistent with HIV incidence findings in other African countries such as Uganda, Mozambique, South Africa, Kenya, Botswana; that higher and new infections were among younger participants [28, 33] The high HIV incidence among women cohabitating with their sexual partners is alarming and supports the use of partner HIV testing and counselling services. The study result is also similar to a study in Mozambique [34] and this calls for extensive HIV prevention efforts such as the use of pre‐exposure prophylaxis (PrEP) in addition to other prevention methods particularly among women with multiple sexual partners. UNAIDS recommends a major movement to protect adolescent girls and young women [28].

Incidence among those who had ever had sex was observed to be 0.46% and zero in those who had never had sex. This demonstrates that the characteristics of HIV epidemics vary. In African countries, HIV is acquired primarily through heterosexual contact and from mother to child. Although key populations contribute to the spread of HIV, heterosexual sex, particularly of low‐risk sex, was reported to make up about 80% of HIV transmission in Nigeria [35]. HIV incidence in the rural areas was slightly higher than the urban areas. Most of the population in Akwa Ibom State are rural dwellers and require more focused HIV programmes. High HIV incidence has also been reported in the rural setting of Botswana [33].

This study presents some limitations because of the cross‐sectional observational design. The number of newly infected HIV participants was relatively small leading to wide and overlapping confidence intervals. There was no baseline FRR available for the country; this could also influence the incidence estimates for the study resulting in a lack of precision and wider confidence interval that was observed. The application of more robust tools for sample size estimation is important [20, 21]and need to establish a baseline FRR for the country for future studies. A reliable HIV incidence estimate requires a large survey sample size. Besides, these tests cannot reliably detect reductions in incidence over time. According to statistical models developed by SACEMA [18] (South African Centre for Epidemiological Modelling and Analysis), results generated using LAg assay may not be precise enough when compared with similar surveys done at a later time. The incidence was calculated based on recent infection assays which may vary according to the different HIV subtypes. Possible misclassifications using standard LAg avidity assay and VL algorithm had been reported in Cameroun [6]. Cameroon has a similar subtype distribution as Nigeria although the circulating viral subtypes may be similar, our study did not determine it. The small population size in the Lynch *et al* study could limit the statistical inference as described by the authors. This could serve as a potential source of misclassification. Recency testing is an imperfect science and there is no gold standard to this. Our study used MDRI of 130 days based on the developer of the assay. MDRI depends on the “diagnostic delay” of the screening test. Values cited in the literature are based, explicitly or implicitly, on a specific algorithm for defining “detectable infection” of which recent infection is a subset. It also depends on HIV‐subtype.

Furthermore, all serology‐based HIV incidence assays are subject to some degree of misclassification, typically because of innate immune variation, differences in HIV‐1 subtypes and prolonged use of ART [36]. Thus, accurate information on the incidence rate of HIV would have a valuable impact on the response to the global HIV epidemic, however, laboratory methods focused on the biological target (biomarker) in the early phase of infection, coupled with an individual variation that may have affected its accuracy thereby conflicted with other estimates and epidemiological information [37].

## 5 CONCLUSIONS

HIV is still a major contributor to the burden of disease in Akwa Ibom State and is particularly devastating because it affects the population in their most productive years. The finding of the high HIV incidence among the 15 to 49‐year age group calls for renewed and innovative efforts to prevent HIV infection among young adults. The need to urgently reach the younger generation with HIV prevention and treatment services, and put in place research that can better understand the factors that are driving HIV transmission among adolescents and young adults in Akwa Ibom State.

## COMPETING INTERESTS

Authors have no competing interests to declare.

## AUTHORS’ CONTRIBUTIONS

ORN, OB, OA, TB and KT conceived the study design and research questions. ORN, OB, OA, ID, TB and KT developed the protocol. ORN, OB, OA, TB, AE, HK and KT oversaw study implementation and data collation. KT, ORN and TB conducted data analysis and data interpretation. ORN conducted a literature search and wrote the first draft of the manuscript. ORN, OA, HK, SRP, ID, AE, EAO, EJ, TB, JR, TDM and KT provided a review of the manuscript at different stages of the draft versions. TDM, KT, TB, OA and ORN assisted with a critical review of the manuscript. All authors read and approved the final manuscript.

## Supporting information


**Additional file S1.** HIV Testing Algorithm_Field_Satellite_CQCL

**Additional file S2.** LAg data management file
