# Supplementary material for: Estimating HIV incidence in the Akwa Ibom AIDS indicator survey (AKAIS), Nigeria using the limiting antigen avidity recency assay
Source: J Int AIDS Soc. 2021 Feb 22;24(2):e25669. doi: 10.1002/jia2.25669 (PMC7900440; doi:10.1002/jia2.25669)
Supplement: Supplementary file 1 — Additional file S1. HIV Testing Algorithm_Field_Satellite_CQCL [file JIA2-24-e25669-s001.docx]

Appendix B National HIV testing Algorithm from the field

**House Hold HIV Testing**

**Key**

**A1 = Determine RTK**

**A2 = Unigold RTK**

**A3 = Stat Pak RTK**

**(+) = REACTIVE**

**(-) = NON-REACTIVE**

**A1**

**>18mths >18mths**

**<18mths**

**A1 (-)**

**A1 (+)**

**A1 (+) & A1 (-)**

Collect DBS sample for all & send to CQCL lab for EID

A3

**A1(+) & A2(-)**

A1(+) & A2(-) & A3(-)

Report as Presumptive Negative

A1(+) & A2(-) & A3(+)

Report as Presumptive Positive

A1(+) & A2(+)

Report as Presumptive Positive

**A2**

**A1(-)**

Report as Presumptive Negative

Only Collect 10% of the Presumptive negative for QC at Sat. labs

Determine reactive

A1(+) & A2(+)

Report as Presumptive Positive

Do CD4 test for all positives at Satellite Labs and Send all positives samples (plasma) and discordant’ s from Sat. lab to CQCL lab for Confirmatory test using Geenius testing (B1)

**Confirmatory Final HIV Test Results**

**Key**

**B1 = Geenius HIV 1/2**

B1

B1 (HIV Indeterminate; HIV 1 or HIV 2 Indeterminate

B1 (+ HIV Untypable)

B1 (+ HIV -1)

Confirmed Positive

B1 (- HIV)

Confirmed Negative

B1 (+ HIV-2)

Confirmed Positive

Send for WBLot to confirm HIV type

B1(+ HIV -1)

Confirmed Positive

**Send all confirmed HIV positives and Indeterminates for Diagnostic HIV -1 Viral** **Load Assay (RNA PCR)**

**Send all confirmed HIV -1 positives for Sedia LAg Avidity Assay**

**Confirmatory Final HIV Test Results for Babies <18mths**

**Send all exposed babies DBS samples for Diagnostic HIV -1 DNA** Assay (DNA PCR)

**Key**

**E2 = Roche Cobas HIV-1 DNA PCR kit**

E2

E2 (+ HIV -1)

Confirmed Positive

E2 (- HIV)

Confirmed Negative
